# The Impact of Video-Assisted Debriefing on Fostering Self-Critical Thinking in Mental Health Nursing Students

**DOI:** 10.1155/jonm/5598639

**Published:** 2025-05-09

**Authors:** Álvaro León-Campos, Casta Quemada-González, Laura Gutiérrez-Rodríguez, Bibiana Pérez-Ardanaz, Silvia García-Mayor, Shakira Kaknani-Uttumchandani, Celia Martí-García

**Affiliations:** ^1^Departamento de Enfermería, Facultad de Ciencias de la Salud, Universidad de Málaga, Málaga, Spain; ^2^Instituto de Investigación Biomédica de Málaga y Plataforma en Nanomedicina (IBIMA Plataforma BIONAND), Málaga, Spain; ^3^Departamento de Enfermería, Facultad de Ciencias de la Salud, Universidad de Granada, Granada, Spain

**Keywords:** debriefing, mental health, nursing education, self-assessment, simulation training

## Abstract

**Aim:** To investigate the effect of using recordings derived from interventions in simulated mental health scenarios on the self-critical capacity of nursing undergraduate students.

**Background:** Video-assisted debriefing (VAD) allows students to visualize their intervention, clarifying certain aspects that might be clouded in their memory. It facilitates the application of theoretical knowledge to real practice and the self-critical capacity to detect strengths and weaknesses in the interventions developed. Despite the benefits of this educational resource, no previous studies have been found that analyze the impact of using recordings on the development of self-critical capacity in the field of mental health by nursing students.

**Methods:** An exploratory quasi-experimental study was conducted based on quantitative and qualitative data collected from students after double debriefing sessions (verbal and video-assisted). Quantitative data identified self-critical capacity trends, while qualitative analysis explored in-depth reflections on strengths, weaknesses, and emotional responses. Both datasets were integrated for comprehensive insights.

**Results:** Quantitative data revealed no significant differences between the two self-evaluation moments. However, the qualitative analysis brought a wide range of in-depth reflections and identified strengths, especially during the VAD. In addition, students felt more reflective and critical about their performance when watching the video, highlighting feelings of self-awareness and opportunities to identify more specific areas for improvement.

**Conclusions:** This study underscores the importance of VAD in enhancing nursing students' self-perception of competencies and emotional responses during mental health simulation scenarios. Certain aspects of mental health care remain paramount for students regardless of video review, emphasizing the need for targeted training in these areas. Employing multiple approaches in data collection is essential to obtain a thorough understanding of students' experiences.

**Implications for Nursing Management:** Nursing managers should integrate clinical simulations and VADs into training programs to enhance competency development, reduce stress, and foster a supportive learning environment.

## 1. Introduction

Mental health problems (MHP) are increasingly prevalent globally and represent a leading cause of disability in children and adolescents [1]. The age of onset is often quite early [2], leading to multiple consequences and a reduction in life expectancy for those affected [3]. Early intervention is essential to prevent the emergence of MHP and alleviate symptoms, potentially improving long-term prognosis [3].

Nursing staff have close contact with patients, placing them in a privileged position to act in this regard [4]. However, many professionals report feeling insufficiently prepared to care for people with MHP, including those at risk for suicidal behavior [5].

University curricula must consider these premises when designing their training programs, promoting the acquisition of specific competencies among nursing degree students that enhance their communication skills with patients [6,7].

To address these gaps in training and better prepare nursing students for the complexities of mental health care, video-assisted debriefing (VAD), a method where audiovisual recordings of simulated interventions are used during postsimulation feedback sessions, emerges as a promising educational tool [8,9]. By bridging theoretical knowledge and practical skills, VAD enables students to critically reflect on their performance, fostering self-awareness and competency development in complex scenarios.

### 1.1. Background

The COVID-19 pandemic has exacerbated MHP worldwide, especially issues of anxiety and depression [10] and self-harming attempts [11]. Current healthcare models do not adequately address these issues, highlighting the need for a paradigm shift in mental healthcare practice that includes better training for healthcare staff [12].

Specifically, nursing staff need to develop necessary skills to care for people with MHP in a broad range of settings [13]. This care is a therapeutic process that unfolds through interpersonal relationships of attention and support between the nurse and the patient, addressing different aspects such as self-awareness, empathy, and affective communication [14]. These skills are strongly related to emotional intelligence [15] yet they are poorly addressed in curricula [16]. Nurses identify these abilities as a weakness, making them feel insecure when communicating, especially in challenging scenarios involving MHP (i.e., those that include MHP) [16].

Thus, mental health nursing requires deeper interpersonal and self-reflection skills compared to other nursing specialties. Professionals must establish strong therapeutic relationships, manage emotionally intense situations, and adapt to the complexities of human behavior. These demands heighten the need for robust self-critical capacity to continuously evaluate and improve clinical practice. However, the existing literature does not sufficiently address how to develop this capacity in mental health contexts, highlighting a significant gap in nursing education [17].

Clinical simulation has been identified as a useful tool for acquiring specific competencies, developing confidence and critical thinking, and improving student satisfaction [18–20]. It has already been applied in the field of mental health education, emphasizing its importance for nursing students before joining clinical mental health units, as it could impact the care provided [21].

Within the methodology used, an important aspect for analyzing effectiveness, promoting changes in approach, competency training, and student satisfaction, is the debriefing phase. Observations made by faculty and peers during this phase encourage communication skills and cognitive learning [22], helping students recognize their weaknesses and strengths [23]. The in-depth discussion and critical reflections during this phase significantly benefit students in subjects such as mental health, serving as a complement to clinical rotations that should be incorporated into their curricula [24].

A specific type of debriefing is one in which the facilitator supports the session with the audiovisual material. VAD allows students to visualize their intervention, clarifying aspects that might be clouded in their memory [9], often due to stage fright [25]. It facilitates the application of theoretical knowledge to real practice and the self-critical capacity to detect strengths and weaknesses in the interventions developed [9]. This type of debriefing appears to have advantages over the classic or verbal debriefing, with greater effectiveness in developing critical thinking and improving student experience [26] and satisfaction [27]. The audiovisual material has been used not only to support debriefing but also to improve the acquisition of competencies in nursing students, promoting active observation [28]. This material can aid in learning of cross-curricular competencies, such as communication skills, allowing students to observe their performance and modify it through reflective and critical thinking [29]. The International Nursing Association for Clinical Simulation and Learning (INACSL) proposed that debriefing should occur in multiple phases to allow deeper exploration of learners' performance and thinking process. This should “allow the observation and discussion of the learner's response and/or behavior to improve performance, particularly when the learner is unaware of a deficit” [8]. VAD can offer significant advantages in this regard.

Despite the benefits of this educational resource, no previous studies have analyzed the impact of using recordings on the development of self-critical capacity in the field of mental health by nursing students. Therefore, the objective of this study was to investigate the effect of VAD using recordings from interventions in simulated mental health scenarios on the self-critical capacity of nursing undergraduate students during the debriefing phase. Secondary objectives included comparing students' evaluations with those made by peers and instructors and exploring students' feelings during the case performance and when watching themselves in the video.

## 2. Methods

### 2.1. Design

This exploratory study, with a quasi-experimental design, utilized a combination of data collection methods through Likert scale questionnaires and open-ended questions [30]. A single group of students self-evaluated in two different debriefing sessions: one after the enactment of the case and another after observing the recordings of their interventions. For the collection of quantitative data, an ad hoc structured checklist, specifically designed for this study, was utilized to evaluate students' performance in simulated scenarios. This checklist included items related to best practices in therapeutic relationships and communication, as well as the identification of nonlistening attitudes. The ad hoc design was chosen to address the particular objectives and context of the study. Items were rated on a five-point Likert scale, covering areas such as therapeutic communication, identification of nonlistening attitudes, and the application of nursing best practices.

Simultaneously, qualitative data were collected through an open-ended questionnaire that asked how they felt during the interventions and what strengths and weaknesses, or areas for improvement, they had identified in their performance.

Thus, the quantitative data were analyzed descriptively to identify trends and patterns in the responses, while the qualitative information involved a descriptive content analysis, allowing the identification of emerging themes and patterns in the students' responses. The frequencies with which individual codes appeared were analyzed to explore potential changes in their occurrence between the two debriefing sessions.

Both sets of data were analyzed separately and subsequently integrated during the interpretation phase to provide a more holistic and in-depth understanding of the students' perceptions.

### 2.2. Participants and Setting

All third-year nursing students enrolled in the Mental and Psychiatric Health Nursing II course at the University of Málaga during the 2020/2021 academic year were invited to participate. Of the 152 enrolled students, 149 consented to have their data included in the analysis.

A total of eight simulated scenarios were designed by faculty experts (Ph.D. holders with experience in mental health simulation training, research, and teaching) and peer-reviewed by mental health nurses. These scenarios adhered to the Health Care Simulation Standards of Best Practice set by the INACSL [31] and were developed using the nursing taxonomy from the North American Nursing Diagnosis Association (NANDA), Nursing Diagnoses, Nursing Interventions Classification (NIC), and Nursing Outcomes Classification (NOC) [32–34].

The simulation sessions were led by three instructors: two mental health nurses with extensive clinical experience (one also a psychologist) and a nurse-psychologist with extensive experience in research and educational innovation.

The scenarios took place in the simulation laboratories of the Faculty of Health Sciences. These laboratories were equipped to simulate real-world settings, such as a hospitalization unit, a primary care consultation, an emergency department waiting room, and a home environment. Students had at their disposal materials according to the scenario, including medication, IV stands, gloves, and electrocardiograph.

Each laboratory featured a video recording system, enabling the rest of the group to observe interventions via streaming and allowing for later review during VAD sessions. Patients and family members were portrayed by mental health nurses to ensure realistic role-playing. Before the seminars, students received guidance from team members to prepare for the scenarios.

### 2.3. Description of the Educational Program

The educational program consisted of 12 theoretical sessions focused on essential knowledge about various mental disorders, including their etiology, symptomatology, and treatment, as well as primary nursing interventions.

In preparation for the practical component, an online preseminar session introduced the session dynamics and explained the questionnaires to be completed during the scenarios. Students were divided into small groups of approximately 10 members, each participating in four simulation sessions lasting 2 h each. Each student was required to intervene in one case, addressing conditions such as anxiety, mood and psychotic disorders, risk of suicidal behavior, personality disorders, and eating behavior disorders. These scenarios were designed to reflect nonspecialized environments, including hospitalization units, primary care, and emergency departments, where nursing students are likely to work during their clinical placements and professional career, even if they do not specialize in mental health.

This study employed a double debriefing session structure. The first session used traditional oral debriefing, while the second session incorporated video recordings of the students' interventions. After each intervention, a verbal debriefing was conducted. Upon completing all simulation sessions, students reviewed their recorded interventions in a VAD session. These sessions encouraged students to reflect on their emotional responses, achieved learning objectives, correct actions, areas for improvement, overall satisfaction with the exercise, and any additional insights relevant to their learning process.

Both debriefing sessions followed the Plus-Delta model, a widely used approach in simulation-based education that emphasizes participant self-assessment. This model helps identify positive aspects of performance (“plus”) and areas for improvement (“delta”) [35].

### 2.4. Data Collection

Intentional convenience sampling was used. Data collection occurred at two different times: Time 1, during the simulation session (Stud.A1), and Time 2, when students watched the videos of their interventions (Stud.A2). Both sessions included a structured debriefing with similar characteristics. During Time 1, the questionnaire on best practices in therapeutic relationships and communication, as well as the identification of nonlistening attitudes, was also completed by peers and the instructor.

The data were collected between March and June 2021 as part of the educational innovation project PIE19-039, “Clinical Simulation in the Acquisition of Mental and Psychiatric Health Competencies in Nursing,” funded by the University of Málaga within the call for educational innovation projects 2019–2021.

**Figure 1:**
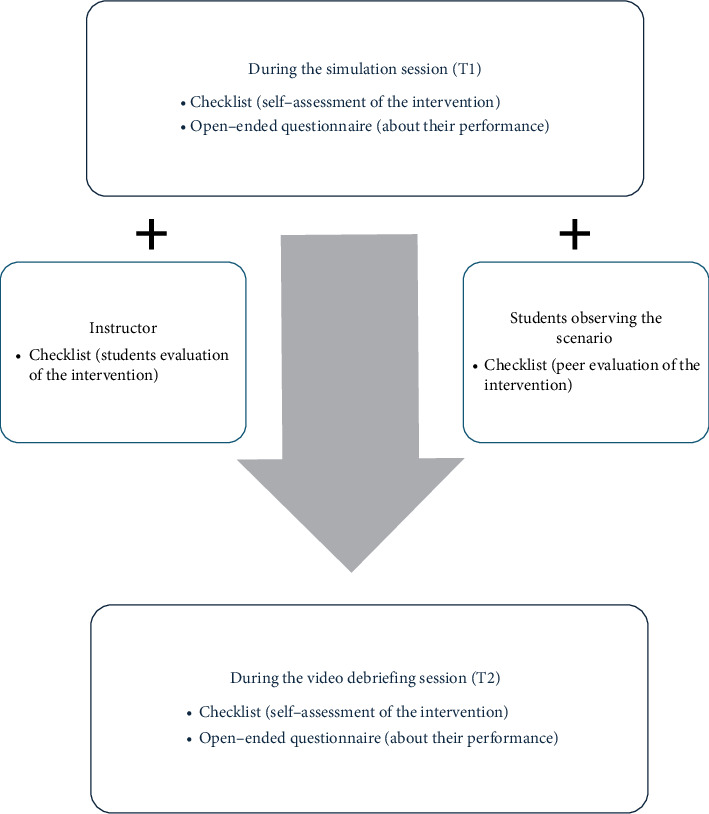
Overview of the data collection procedure.

The procedure to collect the data is summarized as follows (Figure 1).

#### 2.4.1. During the Simulation Session

Intervention Evaluation: All students had a paper form (referred to as a “checklist”) to evaluate their perception of the intervention carried out by the peer who was presenting the case at that moment. This included communication and clinical observation strategies developed by the student during the scenario, with aspects such as empathy and the use of body language, as well as nonlistening attitudes such as ridiculing, blaming, or labeling. The first group was evaluated using a five-point Likert scale (1 = *strongly disagree* and 5 = *strongly agree*), while the second group used a five-point scale to rate the frequency of observed behaviors (1 = *minimum frequency* and 5 = *maximum frequency*). Lastly, an overall assessment of the intervention out of 10 was requested. The students were provided with examples of both communication and listening competencies, as well as nonlistening attitudes, to ensure they clearly understood what they needed to observe. This questionnaire was anonymous to ensure the greatest objectivity in the responses. Students placed all responses face down on a table at the end of the session. This questionnaire was also completed by the professor leading the session.

Self-Assessment of the Intervention: The students participating as nurses in the simulation filled out the same form previously explained (the checklist) but this time about their own intervention (not anonymized). They also completed an open-ended questionnaire with questions about their feelings during the simulation, self-assessment of strengths and areas for improvement, and any additional comment about the seminars.

#### 2.4.2. During the Video Debriefing Session

During the final seminar, students watched recordings of the previously enacted cases, followed by a structured debriefing session. Students completed the same checklist and open-ended questionnaire once again to conduct a self-assessment, reflecting on their interventions after viewing the recorded cases.

Completing the self-assessment after viewing the video allowed students to critically review their interventions with the benefit of hindsight, identifying both strengths and areas for improvement that might have been overlooked during the live simulation. The video provided an opportunity for students to observe nuances in their own behavior, such as body language, tone of voice, and communication style, which are often difficult to evaluate in real time. This process fosters a deeper level of critical reflection, as students are able to analyze their performance more objectively and integrate these insights into their learning experience [26, 36].

### 2.5. Ethics

The study was approved by the research committee of the Faculty of Health Sciences of the University of Málaga and conducted in accordance with the principles of the Declaration of Helsinki. All students enrolled in the course received both verbal and written information regarding the nature of the study. Participation was entirely voluntary, with no implications for the students' academic evaluations. The teaching methodology remained consistent for all enrolled students, regardless of whether they participated in the study. The only difference was that the data from those who gave their consent were included as part of the study. They participated in the scenarios, but participation in the study was optional. They were informed at the beginning of the course, also delivering a consent form that they had to sign if they wanted to participate.

The questionnaires were administered electronically and on paper. An external researcher who was not part of the teaching team was responsible for preparing the anonymized database for analysis. Another individual was responsible for preparing the documents containing the qualitative data for subsequent analysis.

The data were handled confidentially throughout the study, with the anonymization of the previously mentioned questionnaires and the coding of those that required pre- and posttest measures.

All personal data collected during the study were processed in full compliance with the current Spanish data protection laws, specifically the Organic Law 3/2018 on Personal Data Protection and Guarantee of Digital Rights (LOPDGDD) and the General Data Protection Regulation (GDPR) of the European Union.

### 2.6. Analysis

For the quantitative data, descriptive and exploratory analyses were performed to obtain measures of central tendency and dispersion or percentages, depending on the nature of the data. The Kolmogorov–Smirnov test was applied to check the normality of the data. The test showed a nonnormal distribution of the data, so the analyses used were nonparametric tests.

To compare potential differences in the students' self-critical capacity, the Kruskal–Wallis' test was used, comparing the scores of peers with those of the instructors and those of the student themselves at the two moments of their self-assessment (on the day of their intervention and during the viewing of the recordings in the VAD session).

All the analyses were performed with the SPSS v.25 package [37]. All the results were considered statistically significant at *p* < 0.05.

Regarding qualitative data, a content analysis was performed. This approach involved systematically categorizing textual data to identify patterns and themes. The units of analysis comprised the questionnaires completed by the students regarding their perceptions of the actions they performed during the scenario.

Following the inductive analysis of responses to the open-ended questions, the data from the simulation day were compared with the data from the VAD session to identify any changes in the students' reflections and self-critical capacity. The absolute and relative frequencies of each category were calculated in relation to the overall categories. This same method was applied to the subcategories within each category.

Although this study aimed to reflect the authentic perspectives of nursing students, it is important to recognize the intrinsic subjectivity of qualitative research. To minimize potential researcher bias, we adopted a process of methodical reflexivity, ensuring transparency in the analysis process and reporting of findings. Specifically, researchers maintained detailed records of analytical decisions, regularly engaged in discussions to critically examine assumptions, and documented their reflections throughout the research process. This iterative engagement fostered self-awareness and mitigated the risk of bias stemming from preconceived notions or individual experiences. We ensured the study's rigor by utilizing the extensive teaching and research experience of the lecturers, selecting a diverse group of participants across eight different scenarios, and employing self-administered questionnaires to prevent interaction biases. Transferability and applicability were enhanced through detailed descriptions of the context and participants. Reliability and consistency were achieved by having two researchers independently analyze and code the data, followed by a review from two additional researchers [38].

Because of the variety of codes identified, only those with a relative frequency exceeding 5% at either time point were included [39]. The analysis was executed using the ATLAS.ti software version 23.2.1 [40].

The study adheres to the Transparent Reporting of Evaluations with Nonrandomized Designs (TRENDs) guidelines (Supporting File 1).

## 3. Results

### 3.1. Participant Characteristics

Out of the 152 students enrolled in the course, a total of 149 gave their consent to participate in the study and completed the questionnaires. Of these 149 participants, 83.90% were women and the average age was 22.75 years (±6.21).

### 3.2. Intra- and Interobserver Comparison of Student Performance Evaluations

The results of the Kruskal–Wallis analysis comparing the responses regarding student performance revealed that there were no significant differences between the two self-evaluation moments conducted by the students. However, significant overall differences were found in the scores given by peers and the instructor. Peers generally provided higher scores for correctly performed interventions, while the instructor's scores were consistently lower. In terms of overall performance evaluation, peers rated the interventions significantly higher (*H* = 149.415, *p* < 0.001), with a median score of 8.20, compared to the instructor and the self-assessments, both of which were lower and did not differ significantly from each other.

Regarding communication and clinical observation strategies, peers rated the ability to identify oneself significantly higher than both the instructor and the students' self-assessments (*H* = 121.370, *p* < 0.001). This trend was also observed in the scores for promoting responsibility (*H* = 125.364, *p* < 0.001), where the instructor's scores were notably lower compared to those of the students and peers. The use of appropriate nonverbal language also showed significant differences (*H* = 59.606, *p* < 0.001), with peers giving the highest scores (Table 1).

Pairwise comparisons showed no significant differences between the two moments when the students self-assessed (Stud.A1 and Stud.A2). However, there were significant differences for almost all items between the evaluation on the day of the scenario (Stud.A1) and that of the peers, who rated the intervention more positive. In the case of the comparison with the instructor, the differences were reversed, except for the use of appropriate nonverbal language, where no significant differences were found at Stud.A1. When comparing peers with the instructor, peers always rated the intervention more positively. The comparison of the self-assessment on the day of the debriefing (Stud.A2) with peers and the instructor yielded similar results (Supporting File 2).

**Figure 2:**
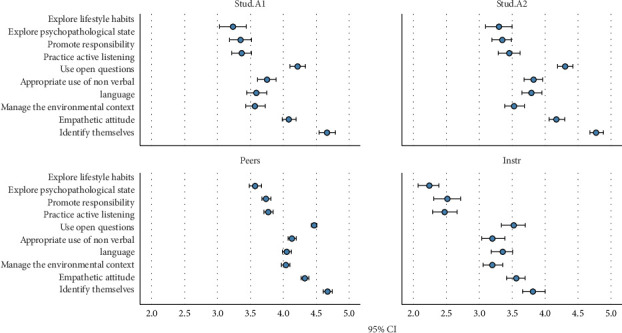
Boxplot of self-assessment, peer, and instructor evaluations of communications and clinical observation strategies.

When comparing self-assessments between Stud.A1 (scenario day) and Stud.A2 (after the VAD), no significant differences in the dispersion of most items were observed (Figure 2). However, items such as “explores psychopathological state” and “promotes responsibility” show slightly greater dispersion in Stud.A2. Peer evaluations (Peers) tended to be higher and less dispersed compared to those of the instructors (Instr).

Nonlistening attitudes showed significant patterns. Peer evaluations revealed significantly higher scores for behaviors such as ordering, directing, or commanding (*H* = 73.588, *p* < 0.001), as well as blaming, ridiculing, or labeling (*H* = 81.747, *p* < 0.001), compared to both instructor evaluations and self-assessments. Peers also found persuading with logic, discussion, or teaching (*H* = 28.903, *p* < 0.001) more problematic compared to the instructor (Table 2).

Regarding the nonlistening attitudes, when comparing the student's self-assessments (Stud.A1 and Stud.A2), pairwise comparisons showed only significant difference for “withdrawing, distracting, joking, and changing the subject,” which was detected more frequently by the students during the VAD. In the comparisons between the self-assessments and the scores given by peers, significant differences were found in all variables, except for “giving premature advice,” “persuading,” and “sympathizing,” in both debriefing sessions. For the remaining variables, peers detected nonlistening attitudes more frequently than the student performing the simulation at both times. However, in the variable “joking or changing the subject” during VAD, this pattern did not hold. In the comparisons between the student's self-assessment evaluations (Sutd.A1 and Stud.A2) and the scores given by the instructor, significant differences were found in six of the 12 items. For “giving premature advice,” “persuading,” “begging,” “interpreting,” and “sympathizing,” the students detected these nonlistening attitudes more frequently than the instructor at both times they completed the questionnaire. In the case of “moralizing,” it was the opposite. Significant differences were found for “joking or changing the subject,” detected more frequently by the students during the VAD. Lastly, in the comparisons between peers and the instructor, significant differences were found in all items except for “moralizing” and “joking.” In all cases, peers indicated a higher frequency of using these nonlistening attitudes (Supporting File 3).

### 3.3. Comparison of Responses to Open-Ended Questions Immediately Postscenario and Postvideo Debriefing

Qualitative analysis of the strengths and weaknesses identified by the students during self-assessment immediately after the scenario and during the VAD revealed three main categories and eight subcategories that offer a more granular perspective on students' strengths and areas for improvement. These categories reflect key dimensions of nursing practice: professional and ethical skills, patient care, and clinical assessment and management, each representing distinct yet complementary aspects of competency development. The subcategories further delineate specific attributes or behaviors that align with these broader categories. This categorization served as the framework for analyzing the data, allowing for a comparison of the frequency and depth of reflections across the two debriefing sessions. Exemplar quotes from students are provided in Table 3 to illustrate these insights.

In terms of overall frequency, professional and ethical skills emerged as the most prominent category, followed by patient care and clinical assessment and management (Table 4). However, differences in the distribution of subcategories within each category highlight unique patterns of reflection between the immediate postscenario assessment and the VAD session.

Overall, students reported more strengths and weaknesses during the VAD session, although the frequencies of reported weaknesses were very similar at both times. However, these figures should be examined in detail by categories and subcategories.

#### 3.3.1. Professional and Ethical Skills

This category includes the competencies related to maintaining integrity, accountability, and adherence to professional standards. It covers ethical decision-making, effective communication, and the establishment of therapeutic relationships. These skills were frequently identified as both strengths and areas for improvement in each debriefing session, with a higher frequency after the VAD.

Among the subcategories that emerged, communication and relational skills was the most frequent. In this case, students identified a greater number of strengths and weaknesses upon reviewing their own performance. Specifically, *Active Listening* was a frequently detected code, especially as a strength after completing the case.

“I actively listened, and I believe he was able to vent about his feelings” [P10_A2]

“I have shown an empathetic attitude, actively listening correctly, showing interest in what the patient was telling me and inquiring about their habits” [P17_A2]

On the other hand, *Effective Communication: Verbal Communication* was the most frequent weakness detected, especially in the case of the VAD.

“Asking more questions related to their problem. Use formal language rather than informal, knowing what I can say and what I shouldn't and, insist less on certain topics. Know in general how to approach patients with mental health problems” [P13_A2]

It should be noted that *Building a Trust Relationship* and *Closeness, r*eferring to behaviors aimed at establishing trust and a sense of emotional and psychological proximity with the patient, were primarily identified as strengths at both times, while *Showing Self-Confidence* only appeared as a weakness in both instances.

The professional development and ethics subcategory was particularly identified as a weakness following the oral debriefing but emerged as a strength after the VAD. The most common codes were related to *Continuing Education* and *Attitude of Tolerance/Respect*. These were primarily noted as areas for improvement after the scenario but were seen as strengths following the video session. Students recognized the importance of enhancing their specialized knowledge in the field of mental health.

“I need to continue training in all aspects to improve my interactions with patients, as well as to have greater theoretical knowledge in relation to all disorders” [P17_A1]

#### 3.3.2. Patient Care

This category encompasses the essential skills and interventions required for providing holistic patient care, focusing on emotional and behavioral management, comprehensive approaches, and effective support and accompaniment. It involves understanding and addressing patients' needs, fostering empathetic attitudes, managing environmental contexts, and prioritizing interventions to ensure well-rounded and compassionate care.

During the simulation, students detected more strengths in the patient care category, specifically within the emotional and behavioral management subcategory. As for weaknesses, the frequency of codes was similar for both groups.

The most frequent subcategory, with almost 60% of the total, was emotional and behavioral management, identifying over twice as many strengths as weaknesses. Students particularly identified *Empathetic Attitude* as a strength, with a slightly higher frequency during the day they completed the scenario.

“I felt her suffering and how she turned to me for help, seeking someone who would listen and understand her” [P5_A1]

The code *Calm Attitude* was identified as both a strength and a weakness with almost the same frequency on the day of the scenario. However, on the day of the VAD, it was reported nearly twice as often as a strength.

“I believe I remained calm even when the patient ‘crossed the line' with me, thus disrespecting me” [P5_A2]

The next subcategory was comprehensive approach, primarily detected as a weakness during the VAD, with the code *Environmental Context Management* leading the list by frequency. It refers to the skills and strategies used by health care professionals to control and optimize the physical and social environment of the patient, including creating a safe and comfortable space. This code was more often identified as a strength when performing the case.

“I tried to calm her down, and took her to a quieter room for a more peaceful environment” [P4_A1]

For the code *Patient-Family Care*, students observed this issue mainly as a weakness during the simulation session. It involves treating the patient in the context of their family dynamics and engaging family members in the care process. Conversely, the code for *Holistic Intervention* was consistently identified as a weakness on both occasions.

“I should have attended to the father's demand regarding the idea of weighing Patricia” [P25_A1]

“I should continue working on the overall approach to the consultation and keep in mind that the role of family members can be a double-edged sword” [P128_A2]

Finally, the subcategory of patient accompaniment and support showed similar frequencies at both times, being detected both as a strength and a weakness. *Considering the Patient's Opinion* was notably high as a strength, primarily during the simulation session. The *Prioritizing* code pertained to the challenge of determining which interventions to prioritize.

“I should keep working on identifying what to focus on in such situations, setting aside my shyness, and better demonstrating my help” [P124_A1]

#### 3.3.3. Clinical Assessment Management

This category encompasses the key skills and strategies for clinically assessing and managing patients, especially in mental health contexts. This category includes aspects such as clinical evaluation, symptom analysis and management, and handling complex clinical situations.

It was primarily observed as a weakness, but strengths were detected more often during the VAD session, particularly in the clinical evaluation and analysis, and mediation and resolution strategies subcategories. Differences were also evident for weaknesses.

The most frequent subcategory was clinical evaluation and analysis. Weaknesses were primarily observed during the VAD session, although this category also appeared almost twice as often as a strength in that same session compared to the simulation day.

The most frequent codes identified as strengths during the video debriefing session were *Focused Observation* and *Exploring Habits*. The latter was also noted as a weakness, alongside with *Psychopathological Exploration*, which was nearly twice as frequent compared to the simulation day, both as a strength and as a weakness.

“I feel that I don't handle it appropriately, as there are many aspects of their psychopathological condition that I do not explore, which would be suitable for offering the help they need” [P103_A2]

*Exploring Habits* was identified as a weakness five times during VAD.

The clinical and therapeutic management subcategory primarily appeared as an area for improvement. The code *Situation Management* stood out on the simulation day as a weakness or aspect needing improvement.

The code for *Managing Delusional Thoughts* that appeared on the simulation day was noted as a weakness.

*Time Management*, referring to the time spent in consultation or with the patient, was reported only on the day of the VAD as an area for improvement.

“Primarily, I need to improve my time management and allow more time for the patient to express themselves” [P45_A2]

The resolution and mediation strategies subcategory was perceived as a weakness during the scenario but as a strength during the VAD.

“My ability to solve the problem that arose and to sit next to the patient, showing closeness while respecting her personal space” [P39_A2]

“How to solve patients' problems on my own, considering all variables” [P27_A1]

### 3.4. Emotional Reactions During Simulation and Video Debriefing

In this section, we explored the secondary objective of the study: understanding how students felt during the simulation and when they watched themselves in the VAD.

The responses were coded and categorized into emotions related to extrinsic and intrinsic factors, also distinguished between positive and negative emotions. Intrinsic factors referred to the students' own characteristics or actions, while extrinsic factors were related to the context and the design of the simulation itself.

In general, more positive emotions than negative ones were reported, with 519 (62.83%) and 307 (37.17%), respectively. When comparing the two moments, positive emotions were slightly more frequent during the VAD (*n* = 264; 50.87%). However, negative emotions were more frequent when the students finished their participation in the scenario (*n* = 172; 56.03%).

When comparing by the nature of the factors involved in the emotions, emotions related to intrinsic factors were more frequent (80.02%), with positive emotions predominating both for intrinsic and extrinsic factors (Table 5).

#### 3.4.1. Emotions Related to Intrinsic Factors

Regarding positive emotions, they were more frequent on the VAD session. However, the code that appeared most frequently was *Progressive Adaptation*, which referred to how the student could relax as the case progressed and go with the flow of the events, more present at the end of the simulation. Following this, personal self-reflection, appeared up to three times more frequently in the VAD session.

“I noticed both the things I did well and the mistakes I made. Now I know what proper conduct should be” [P3_A2]

Other notable differences include greater *Satisfaction* and a sense of *Competence* reported after watching the video.

Negative emotions were reported specially after the scenario with the code *Nervousness* predominating. However, when students watched their intervention, they realized that, despite feeling nervous, it was not noticeable. In addition, after the VAD, there was a slight increase in the *Perception of Inefficacy* reported by the students.

“While performing it, I felt nervous, though it is true that it doesn't show as much when viewed from the outside. I also felt that I had not been able to demonstrate my knowledge about the presented health problem and had to deal with a common situation that I did not expect” [P39_A2]

#### 3.4.2. Emotions Related to Extrinsic Factors

Some students mentioned emotions related to external factors or those beyond their control. Positive emotions were more frequently reported on the day they watched their intervention, particularly noting the high occurrence of the *Positive Revaluation* code. This code captures the positive aspect of having the opportunity to see their intervention and reassess their performance, realizing they executed several aspects correctly. This realization allows them to approach these experiences with a more positive outlook.

“After watching the video, I see that certain aspects are not as bad as I perceived them during the seminar, and there are some aspects I didn't pay attention to then but have noticed now” [P18_A2]

However, other codes related to the simulation itself, such as *Realistic Immersion*, *Appreciation* of the simulation, *Feeling Supported*, or the experience *Exceeding their Expectations*, were higher in all cases at the time of performing the scenario.

Negative emotions were more frequent on the day the scenario was developed, although the most frequent code, which was *Feeling Observed*, had a higher frequency on the day the students were able to watch their intervention, together with other similar feelings as *Shame*. The perception of *Lacking Time* to develop the desired interventions was the second most frequent code, appearing more than twice as often on the day of the scenario. As for the *Feeling of Difficulty* and the *Influence of The Simulated Environment*, these were higher on the day of the VAD, especially the latter code.

### 3.5. Integration of Quantitative and Qualitative Findings

The integration of quantitative and qualitative findings highlights complementary insights into the students' self-critical capacity. While quantitative data indicated no significant differences between self-assessments at different time points, the qualitative analysis revealed a richer and more nuanced perspective. Specifically, the qualitative findings emphasized self-awareness and emotional engagement during VAD, which were not fully captured by quantitative measures.

## 4. Discussion

This study aimed to assess the impact of VAD on nursing students' self-perception of competencies and their emotional responses. The findings indicate that while there were no significant differences in quantitative self-assessments scores between the simulation day and the VAD, the qualitative data revealed different aspects of reflection during both sessions. Specifically, during the simulation session, there was a greater emphasis on the need for continued training while students focused on effective communication, challenges in managing the environmental context, exploring habits, and conducting psychopathological assessments during the VAD. These results suggest that VAD allows them to observe aspects of their performance they might not have been aware of during the scenario itself, despite both sessions involving similarly structured debriefings.

Previous research highlights the benefits of VAD in nursing education. Studies have shown that this resource promotes deeper reflection and self-assessment among students by allowing them to observe and critique their own performance, which they might not fully appreciate during live scenarios [41]. Specifically, the ability to view their actions helps students identify areas for improvement that are not immediately evident [42, 43], such as nuances in nonverbal communication, effective management of the environment, and detailed patient assessments.

Results from the analysis of the checklist scores showed no significant differences between the two self-assessments, suggesting that students' perceptions of their competencies were consistent across both time points. This consistency could indicate that the initial simulation provided a robust foundation for self-assessment. Overall, the data suggest a clear trend where peer evaluations tend to be more positive compared to those of the instructor and self-assessments. Peer evaluations also appear less dispersed compared to those of the instructors, indicating a more positive and homogeneous perception. This suggests that peers evaluate more uniformly and favorably while instructors present greater variability and a more critical assessment of the students' performance. This discrepancy could be attributed to the supportive role peers play in an educational setting, contrasting with the instructor's evaluative role. Previous studies have revealed that students themselves could believe that their peers do not provide real and honest feedback, as they tend to be kind and lenient [44]. Even with an anonymous method, students tend to be less inclined to identify weaknesses, fearing it might negatively impact their peers' evaluations [45].

The content analysis of the qualitative data reveals different aspects of reflection during both sessions, allowing us to detect differences not evident in the quantitative data. The detailed analysis of categories and subcategories showed differences in the strengths and weaknesses detected at each debriefing session. It brings a deeper reflection on each aspect and the opportunity to uncover other skills, problems, and even feelings experienced by the students. Themes such as increased self-awareness and critical thinking emerged during the VAD, with students reporting greater emotional engagement when reviewing their recorded performance. This type of reporting provided a deeper perspective, allowing students not only to specify aspects related to competencies and strategies they employed but also to explain their feelings in depth. The importance of utilizing multiple methods in data collection is well-supported by various studies, highlighting the benefits of such an approach in providing a more complete picture of the research problem [46]. Using open-ended questions in research allows for the identification of data that may be omitted or distorted in structured questionnaires. This approach can capture insights into less common aspects and provide a richer context for understanding the participant's perspective. Such details are often missed when respondents are restricted to predefined items, which, although potentially more reliable for data analysis, might yield less valid information [47]. Open-ended questions enable respondents to elaborate on their experiences and thoughts, thereby uncovering valuable information that might otherwise be overlooked.

The analysis of the frequency of codes revealed the importance that students attributed to professional and ethical skills, especially communication and relational abilities. These competencies were frequently identified as both strengths and weaknesses in both evaluation moments, accounting for nearly half of the codes in each case. This emphasis highlights the critical role that effective communication and relationship-building play in nursing practice. It underscores the need for continuous improvement in these areas to enhance patient care and professional development. The recognition of these skills as pivotal reflects their subjective value to the students, reinforcing their significance in nursing education. Unlike other scenarios, where high fidelity plays a crucial role, mental health scenarios fundamentally rely on interpersonal skills, including communication skills [48], which are essential for nursing professionals [49].

The results also highlight the importance of holistic and comprehensive patient care, as shown by the similar frequencies of the categories “Patient Care” and “Clinical Assessment and Management.” The significant identification of emotional and behavioral management skills underscores their crucial role in nursing practice. Students consistently emphasized the importance of an empathetic attitude, reinforcing the need for effective communication and emotional intelligence in improving patient care outcomes. Understanding the relationship between nurses' attitudes toward communication, empathy, and emotional intelligence is essential for enhancing quality of care, patient satisfaction, patient safety, work environment, and professional development [48, 49].

Moreover, it is evident that while certain critical issues such as management of hallucinations, delusional ideas, therapeutic follow-up, treatment adherence, conflict resolution, and boundary setting are less frequently identified by students, they remain pivotal challenges in clinical practice for nurses working with individuals experiencing MHP. These challenges significantly impact the management and prognosis of mental disorders. Effective management of these issues is crucial, as inadequate handling can lead to poorer patient outcomes and increased health care costs [50, 51]. Therefore, incorporating targeted training and support in these areas within nursing education programs is essential for improving clinical practice and patient care outcomes. This observation aligns with Kameg, Fradkin, and Lee [52] who underscore the importance of incorporating realistic patient simulation scenarios to enhance nursing students' competencies and attitudes toward psychiatric nursing. The study revealed that although standardized patient simulations can improve students' satisfaction and confidence in handling MHP, gaps remain in fully addressing the complexities of therapeutic follow-up and conflict resolution. This highlights the necessity for ongoing educational interventions and support mechanisms to better prepare nursing students for the multifaceted demands of mental health care. Continuous exposure and targeted training in these areas are essential to ensure that future nurses can deliver comprehensive and effective care, ultimately improving patient outcomes and advancing mental health practice.

Some previous studies have conducted double debriefing sessions to work on independent disciplines and multidisciplinary teams [53] or to focus on aspects related to patient outcomes and teamwork [54]. In our case, a double session was conducted combining oral debriefing with VAD to enhance the students' reflection and critical thinking process. This approach likely contributed to the predominance of positive emotions reported during the VAD session, encompassing both specific feelings, such as satisfaction and competence, and a general sense of progress and constructive self-assessment. This shift could be because of the opportunity for students to reflect on their performance and identify strengths they might not have noticed initially. This “positive revaluation” helps them recognize their achievements and approach future scenarios with a more constructive mindset. Previous studies have identified VAD sessions as a valuable method to enhance both technical and nontechnical competencies, including communication skills, improving critical thinking, situational awareness, and teamwork abilities [9, 27].

It was also noted that during the VAD, students more frequently felt observed and ashamed. This is understandable considering that during the simulation scenario, they were alone with their patients in a room, whereas, during the VAD session, they were watching the recording with their peers and the instructor. This may have made them feel exposed to criticism despite the positive aspects identified in being able to observe and evaluate their intervention [36]. However, there were no differences in the nervousness caused by this, consistent with findings from other studies that have compared VAD to traditional debriefing methods and reported no significant differences in perceived stress [55].

It is known that students often experience anxiety and lack of confidence when communicating with patients diagnosed with psychiatric illnesses. Understanding how students feel when facing these scenarios is crucial, as many situations can present challenges. Knowing their emotions can help implement appropriate educational strategies that support the acquisition of competencies [7]. Standardized patient simulations in psychiatric nursing provide a controlled environment for students to practice and become proficient in communication skills, mitigating some of these feelings of anxiety, fear, and lack of confidence [56]. These simulations allow students to receive timely and relevant feedback on their clinical performance, further enhancing their confidence and satisfaction in handling psychiatric cases [52]. The key lies in combining these simulated scenarios with a respectful VAD experience that fosters an environment of acceptance for constructive criticism while enhancing learning [36].

The integration of quantitative and qualitative data in this study provides a comprehensive perspective on the development of students' self-critical capacity. While quantitative measures revealed consistent self-perceptions across time points, the qualitative findings offered deeper insights into emotional engagement and self-awareness during VAD. This divergence between datasets highlights the importance of employing mixed-methods approaches, as they allow researchers to capture both measurable outcomes and the complex subjective processes underlying learning experiences [57]. By leveraging these complementary methodologies, this study underscores the need for a holistic understanding of how nursing students develop critical competencies in mental health scenarios.

### 4.1. Limitations and Implications for Research

It's important to recognize certain limitations present in this study. Initially, the use of a small sample of undergraduate nursing students might reduce how applicable our results could be across different groups and settings. In addition, the reliance on self-reports and peer reports may have introduced subjectivity and potential inaccuracies into our findings. It could have led to social desirability bias in the students' responses. When self-assessing and being evaluated by peers and instructors, students might have responded in ways they believed would be viewed favorably, rather than being completely honest. This bias could have affected both the quantitative and qualitative data. Future research could consider using methods to minimize this effect, such as anonymous evaluation or employing triangulation methods to validate the findings.

Furthermore, there was a lack of control for other influential factors such as the participants' prior experiences, their motivation and personality traits, or the impact of feedback from instructors, which could affect both performance and self-assessments. The influence of participants' prior simulation experiences could have affected their performance and reflections during the debriefing sessions. Students with more extensive simulation exposure might exhibit greater familiarity with the process, potentially influencing their engagement and responses. To counter these limitations, forthcoming studies should consider accounting for such prior experiences to better isolate the specific effects of VAD, incorporating larger, more varied participant groups, employing more objective measurements, and designing studies with a control group to ensure a more robust research methodology.

The use of ad hoc designed questionnaires can also be a limitation, as these tools may lack the established validity and reliability of standardized instruments. Their tailored nature might introduce biases or fail to capture the full scope of the variables intended to be measured. This can potentially affect the consistency and generalizability of the findings. Future research should aim to utilize or develop more standardized and validated measurement tools to ensure that the results are robust and comparable across different studies and settings.

An additional limitation of this study is the lack of long-term follow-up, which prevents an evaluation of whether the benefits observed from VAD, such as enhanced self-critical capacity and emotional engagement, translate into sustained behavioral changes or improved clinical outcomes. Future research should explore longitudinal designs to address this gap and provide insights into the enduring impact of VAD on nursing students' competencies.

Despite the limitations of our study and the extensive literature on the benefits of VAD in nursing education, no previous studies have focused on exploring the potential effects of this type of debriefing in specific mental health scenarios. This area of research could provide valuable insights into improving nursing education in mental health contexts, highlighting the need for future studies to address this gap.

### 4.2. Implications for Education and Practice

This research could have important implications for the instruction and practice in mental health nursing. It underscores the necessity of enhancing students' self-efficacy and self-assessment abilities, emphasizing the value of receiving comprehensive, instructive feedback from both educators and fellow learners to advance clinical abilities and knowledge. The findings also encourage a reflection on nurse training curricula, advocating for the integration of pedagogical and assessment methods that promote the cultivation of clinical proficiency and the capacity for critical thinking among aspiring mental health nurses.

The use of VAD provides a unique platform for students to analyze and refine their approaches to challenging psychiatric scenarios that are often difficult to explore fully in traditional clinical settings. For example, reviewing video recordings enables students to identify specific aspects of their communication styles, decision-making processes, and emotional responses during patient interactions. This method can also facilitate in-depth discussions about complex cases, such as managing patients with severe MHP or navigating ethical dilemmas, in a controlled and supportive environment. By offering these opportunities for reflection and targeted feedback, VAD bridges the gap between theoretical knowledge and practical application, equipping students with the tools needed to address the multifaceted demands of mental health nursing.

## 5. Conclusion

This study underscores the importance of VAD in enhancing nursing students' self-perception of competencies, emotional engagement, and reflective capacity during mental health simulation scenarios. While quantitative self-assessments remained consistent, qualitative analysis revealed deeper reflections on effective communication, environmental management, and psychopathological assessments during video reviews. These findings highlight the added value of integrating emotional insights into the self-assessment process. This approach helps students recognize and address their emotional engagement with challenging scenarios, a critical skill in mental health practice.

Notably, certain aspects of mental health care, such as emotional and behavioral management, remain paramount for students regardless of video review, emphasizing the need for targeted training in these areas. VAD enables a more profound engagement with these complexities, offering students a structured environment to develop confidence in navigating emotionally demanding situations and applying reflective thinking. The consistency in self-assessment scores between the simulation day and VAD suggests that the initial simulation experience provides a solid foundation for self-assessment. However, qualitative differences observed between the two debriefing sessions highlight the added depth of reflection and critical thinking that VAD can foster.

These findings highlight the need for incorporating multiple data collection methods to capture a comprehensive view of students' experiences and learning processes. Future research should explore larger, more diverse samples and standardized tools to further validate these findings and enhance nursing curricula to better prepare students for clinical practice in mental health settings.

## Figures and Tables

**Table 1:** Comparison of communication and clinical observation strategies by evaluators (peers, instructor, self-assessment during simulation, and self-assessment during video debriefing).

Communication and clinical observation strategies	Group	Median	IQR	Min	Max	*H*	*p*
Identify themselves	Stud.A1	5.00	0.00	1.00	5.00	121.370	< 0.001
	Stud.A2	5.00	0.00	1.00	5.00		
	Peers	4.80	0.200	2.80	5.00		
	Instructor	4.00	2.00	1.00	5.00		

Empathetic attitude	Stud.A1	4.00	1.00	2.00	5.00	100.373	< 0.001
	Stud.A2	4.00	1.00	2.00	5.00		
	Peers	4.40	0.30	3.40	4.90		
	Instructor	4.00	1.00	2.00	5.00		

Manage the environmental context	Stud.A1	3.00	1.00	1.00	5.00	71.746	< 0.001
	Stud.A2	4.00	1.00	1.00	5.00		
	Peers	4.10	0.50	2.10	4.80		
	Instructor	3.00	1.00	1.00	5.00		

Appropriate use of nonverbal language	Stud.A1	4.00	1.00	1.00	5.00	59.606	< 0.001
	Stud.A2	4.00	1.00	1.00	5.00		
	Peers	4.10	0.60	2.70	4.80		
	Instructor	3.00	1.00	1.00	5.00		

Use open questions	Stud.A1	4.00	1.00	1.00	5.00	84.151	< 0.001
	Stud.A2	4.00	1.00	2.00	5.00		
	Peers	4.20	0.50	2.70	4.90		
	Instructor	3.00	2.00	1.00	5.00		

Practice active listening	Stud.A1	4.00	1.00	2.00	5.00	76.877	< 0.001
	Stud.A2	4.00	1.00	2.00	5.00		
	Peers	4.50	0.40	3.30	5.00		
	Instructor	3.00	1.00	1.00	5.00		

Promote responsibility	Stud.A1	3.00	1.00	1.00	5.00	125.364	< 0.001
	Stud.A2	4.00	1.00	1.00	5.00		
	Peers	3.80	0.60	2.70	4.60		
	Instructor	2.00	1.00	1.00	5.00		

Explore psychopathological state	Stud.A1	3.00	1.00	1.00	5.00	90.269	< 0.001
	Stud.A2	3.00	1.00	1.00	5.00		
	Peers	3.80	0.50	2.30	4.60		
	Instructor	2.00	3.00	1.00	5.00		

Explore lifestyle habits	Stud.A1	3.00	2.00	1.00	5.00	116.911	< 0.001
	Stud.A2	3.00	1.00	1.00	5.00		
	Peers	3.60	0.80	2.10	4.90		
	Instructor	2.00	2.00	1.00	5.00		

Overall performance evaluation	Stud.A1	7.00	1.00	3.00	10.0	149.415	< 0.001
	Stud.A2	7.00	2.00	4.00	10.0		
	Peers	8.20	0.70	5.90	9.40		
	Instructor	6.00	2.00	4.00	10.0		

*Note:* Post hoc Bonferroni analysis *p* < 0.05. Stud.A1 = self-assessment during the simulation session. Stud.A2 = self-assessment during the video debriefing.

**Table 2:** Comparison of nonlistening attitudes by evaluators (peers, instructor, self-assessment during simulation, and self-assessment during video debriefing).

Nonlistening attitudes	Group	Median	IQR	Min	Max	*H*	*p*
Ordering, directing, or commanding	Stud.A1	1.00	1.00	1.00	4.00	73.588	< 0.001
	Stud.A2	1.00	1.00	1.00	5.00		
	Peers	1.50	0.50	1.00	2.80		
	Instructor	1.00	0.00	1.00	4.00		

Threatening	Stud.A1	1.00	0.00	1.00	4.00	86.008	< 0.001
	Stud.A2	1.00	0.00	1.00	4.00		
	Peers	1.10	0.20	1.00	1.70		
	Instructor	1.00	0.00	1.00	3.00		

Giving premature advice, suggestions, or solutions	Stud.A1	3.00	2.00	1.00	5.00	84.045	< 0.001
	Stud.A2	3.00	2.00	1.00	5.00		
	Peers	3.20	0.60	2.10	4.30		
	Instructor	2.00	2.00	1.00	5.00		

Persuading with logic, discussion, or teaching	Stud.A1	3.00	2.00	1.00	5.00	28.903	< 0.001
	Stud.A2	2.00	2.00	1.00	5.00		
	Peers	2.60	0.50	1.60	3.50		
	Instructor	1.00	2.00	1.00	5.00		

Moralizing or preaching	Stud.A1	1.00	0.00	1.00	5.00	80.037	< 0.001
	Stud.A2	1.00	0.00	1.00	4.00		
	Peers	1.30	0.40	1.00	2.20		
	Instructor	1.00	2.00	1.00	5.00		

Disagreeing, judging, and/or criticizing	Stud.A1	1.00	0.00	1.00	5.00	95.430	< 0.001
	Stud.A2	1.00	0.00	1.00	4.00		
	Peers	1.10	0.20	1.00	1.70		
	Instructor	1.00	0.00	1.00	4.00		

Begging	Stud.A1	2.00	2.00	1.00	5.00	56.963	< 0.001
	Stud.A2	2.00	2.00	1.00	5.00		
	Peers	2.10	0.40	1.40	3.50		
	Instructor	1.00	1.00	1.00	4.00		

Blaming, ridiculing, or labeling	Stud.A1	1.00	0.00	1.00	4.00	81.747	< 0.001
	Stud.A2	1.00	0.00	1.00	3.00		
	Peers	1.00	0.10	1.00	1.50		
	Instructor	1.00	0.00	1.00	4.00		

Interpreting or analyzing	Stud.A1	2.00	2.00	1.00	5.00	78.035	< 0.001
	Stud.A2	2.00	2.00	1.00	5.00		
	Peers	2.30	0.40	1.50	3.40		
	Instructor	1.00	1.00	1.00	4.00		

Reaffirming, sympathizing, or consoling	Stud.A1	3.00	3.00	1.00	5.00	109.444	< 0.001
	Stud.A2	3.00	3.00	1.00	5.00		
	Peers	2.80	0.50	1.90	3.80		
	Instructor	1.00	1.00	1.00	4.00		

Questioning or testing	Stud.A1	1.00	0.00	1.00	4.00	162.372	< 0.001
	Stud.A2	1.00	0.00	1.00	4.00		
	Peers	1.30	0.20	1.00	2.20		
	Instructor	1.00	0.00	1.00	4.00		

Withdrawing, distracting, joking, or changing the subject	Stud.A1	1.00	0.00	1.00	4.00	105.626	< 0.001
	Stud.A2	1.10	0.30	1.00	1.70		
	Peers	1.00	0.00	1.00	4.00		
	Instructor	1.00	0.00	1.00	4.00		

*Note:* Post hoc Bonferroni analysis *p* < 0.05. Stud.A1 = self-assessment during the simulation session. Stud.A2 = self-assessment during the video debriefing.

**Table 3:** Categories and subcategories as results of qualitative data analysis.

Categories	Subcategories
Professional and ethical skills	Communication and relational skills
	Professional development and ethics

Clinical assessment and management	Clinical evaluation and analysis
	Clinical and therapeutic management
	Resolution and mediation strategies

Patient care	Emotional and behavioral management
	Comprehensive approach
	Patient accompaniment and support

**Table 4:** Absolute and relative frequencies of strengths and weaknesses identified by students at the end of the simulation and after video-assisted debriefing.

	Strengths Stud.A1		Strengths Stud.A2		Weaknesses Stud.A1		Weaknesses Stud.A2		Total	
	c/TC	%	c/TC	%	c/TC	%	c/TC	%	c/TC	%
Category: professional and ethical skills	*242/450*	*53.78*	*263/487*	*54.00*	*178/381*	*46.72*	*180/385*	*46.75*	*863/1703*	*50.68*
Subcategory: communication and relational skills	232/242	95.87	241/263	91.63	148/178	83.15	163/180	90.56	784/863	90.85
Effective communication: verbal communication	28/232	12.07	38/241	15.77	42/148	28.38	56/163	34.36	164/784	20.92
Active listening	72/232	31.03	62/241	25.73	10/148	6.76	12/163	7.36	156/784	19.90
Effective communication: nonverbal communication	29/232	12.50	26/241	10.79	31/148	20.95	20/163	12.27	106/784	13.52
Building a trust relationship	18/232	7.76	24/241	9.96	2/148	1.35	4/163	2.45	48/784	6.12
Closeness	22/232	9.48	11/241	4.56	2/148	1.35	4/163	2.45	39/784	4.97
Contact	10/232	4.31	19/241	7.88	1/148	0.68	7/163	4.29	37/784	4.72
Showing self-confidence	0/232	0.00	1/241	0.41	18/148	12.16	15/163	9.20	34/784	4.34
Subcategory: professional development and ethics	10/242	4.13	22/236	9.32	30/178	16.85	17/180	9.44	79/863	9.45
Continuing education	0/10	0.00	0/22	0.00	18/30	60.00	7/17	41.18	25/79	31.65
Attitude of tolerance/respect	4/10	40.00	15/22	68.18	1/30	3.33	4/17	23.53	24/79	30.38
Acting professionally	0/10	0.00	0/22	0.00	9/30	30.00	5/17	29.41	14/79	17.72
Effective application of knowledge	5/10	50.00	1/22	4.55	0/30	0.00	0/17	0.00	6/79	7.59
Sincerity	0/10	0.00	2/22	9.09	0/30	0.00	1/17	5.88	3/79	3.80
Promoting responsibility	0/10	0.00	2/22	9.09	0/30	0.00	0/17	0.00	2/79	2.53
Maintaining patient privacy	1/10	10.00	0/22	0.00	0/30	0.00	0/17	0.00	1/79	1.27

*Category: patient care*	*145/450*	*32.22*	*133/487*	*27.31*	*92/381*	*24.15*	*91/385*	*23.64*	*461/1703*	*27.07*
Subcategory: emotional and behavioral management	102/145	70.34	84/133	63.16	45/92	48.91	34/91	37.36	265/461	57.48
Empathetic attitude	53/102	51.96	48/84	57.14	6/45	13.33	8/34	23.53	115/265	43.40
Calm attitude	28/102	27.45	22/84	26.19	27/45	60.00	12/34	35.29	89/265	33.58
Anxiety management	13/102	12.75	12/84	14.29	10/45	22.22	11/34	32.35	46/265	17.36
Subcategory: comprehensive approach	26/145	17.93	31/133	23.31	28/92	30.43	39/91	42.86	124/461	26.90
Environmental context management	16/26	61.54	13/31	41.94	11/28	39.29	21/39	53.85	61/124	49.19
Patient-family care	9/26	34.62	15/31	48.39	5/28	17.86	7/39	17.95	36/124	29.03
Holistic intervention	0/26	0.00	0/31	0.00	5/28	17.86	7/39	17.95	12/124	9.68
Risk management	1/26	3.85	3/31	9.68	5/28	17.86	3/39	7.69	12/124	9.68
Subcategory: patient accompaniment and support	17/145	11.72	18/133	13.53	19/92	20.65	18/91	19.78	72/461	15.62
Considering the patient's opinion	10/17	58.82	6/18	33.33	6/19	31.58	6/18	33.33	28/72	38.89
Accompaniment	7/17	41.18	10/18	55.56	4/19	21.05	2/18	11.11	23/72	31.94
Prioritizing	0/17	0.00	2/18	11.11	9/19	47.37	9/18	50.00	20/72	27.78

*Category: clinical assessment and management*	*63/450*	*14.00*	*91/487*	*18.69*	*111/381*	*29.13*	*114/385*	*29.61*	*379/1703*	*22.25*
Subcategory: clinical evaluation and analysis	35/63	55.56	61/91	67.03	54/111	48.65	76/114	66.67	226/379	59.63
Focused observation	12/35	34.29	22/61	36.07	23/54	42.59	25/76	32.89	82/226	36.28
Exploring habits	4/35	11.43	13/61	21.31	11/54	20.37	20/76	26.32	48/226	21.24
Psychopathological exploration	9/35	25.71	9/61	14.75	9/54	16.67	17/76	22.37	44/226	19.47
Exploring suicide	1/35	2.86	3/61	4.92	6/54	11.11	5/76	6.58	15/226	6.64
Exploring relationships	4/35	11.43	3/61	4.92	2/54	3.70	0/76	0.00	9/226	3.98
Evaluating emotional state	3/35	8.57	9/61	14.75	2/54	3.70	4/76	5.26	18/226	7.96
Subcategory: clinical and therapeutic management	19/63	30.16	16/91	17.58	34/111	30.63	27/114	23.68	96/379	25.33
Situation management	12/19	63.16	12/16	75.00	20/34	58.82	15/27	55.56	59/96	61.46
Protocol compliance	2/19	10.53	0/16	0.00	4/34	11.76	4/27	14.81	10/96	10.42
Managing hallucinations	0/19	0.00	1/16	6.25	2/34	5.88	2/27	7.41	5/96	5.21
Managing delusional thoughts	0/19	0.00	1/16	6.25	4/34	11.76	0/27	0.00	5/96	5.21
Stabilizing the patient	2/19	10.53	1/16	6.25	1/34	2.94	0/27	0.00	4/96	4.17
Knowing available resources	0/19	0.00	0/16	0.00	2/34	5.88	2/27	7.41	4/96	4.17
Treatment adherence	2/19	10.53	0/16	0.00	0/34	0.00	1/27	1.10	3/96	3.13
Time management	0/19	0.00	0/16	0.00	0/34	0.00	3/27	11.11	3/96	3.13
Therapeutic follow-up	0/19	0.00	1/16	6.25	0/34	0.00	0/27	0.00	1/96	1.04
Subcategory: resolution and mediation strategies	9/63	14.29	14/91	15.38	23/111	20.72	11/114	9.65	57/379	15.04
Problem-solving ability	3/9	33.33	8/14	57.14	13/23	56.52	5/11	45.45	29/57	50.88
Searching for alternatives	1/9	11.11	1/14	7.14	4/23	17.39	4/11	36.36	10/57	17.54
Conflict mediation	2/9	22.22	1/14	7.14	3/23	13.04	2/11	18.18	8/57	14.04
Shared decision-making	2/9	22.22	4/14	28.57	2/23	8.70	0/11	0.00	8/57	14.04
Setting boundaries	1/9	11.11	0/14	0.00	0/23	0.00	0/11	0.00	1/57	1.75

*Note:* The table shows the number of times that each code was cited resulting from the analysis of each category concerning the total number of codes in each category or subcategory as appropriate. Stud.A1 = self-assessment during the simulation session. Stud.A2 = self-assessment during the video debriefing.

Abbreviations: c = code and TC = total codes.

**Table 5:** Absolute and relative frequencies of emotions reported in both sessions.

	Stud.A1 *n* = 142		Stud.A2 *n* = 142		Total *N* = 142	
	c/TC	%	c/TC	%	c/TC	%
*Emotions related to intrinsic factors*	*352/427*	*82.44*	*309/399*	*77.44*	*661/826*	*80.02*
Positives	203/352	57.67	194/309	62.78	397/661	60.06
Progressive adaptation	63/203	31.03	27/194	13.92	90/397	22.67
Personal self-reflection	10/203	4.93	39/194	20.10	49/397	12.34
Comfort	29/203	14.29	14/194	7.22	43/397	10.83
Overall positivity	17/203	8.37	13/194	6.70	30/397	7.56
Calmness	16/203	7.88	13/194	6.70	29/397	7.30
Satisfaction	6/203	2.96	20/194	10.31	26/397	6.55
Competent	8/203	3.94	16/194	8.25	24/397	6.05
Self-confidence	6/203	2.96	11/194	5.67	17/397	4.28
Desire for improvement	4/203	1.97	11/194	5.67	15/397	3.78
Negatives	149/352	42.33	115/309	37.22	264/661	39.94
Nervousness	50/149	33.56	26/115	22.61	76/264	28.79
Perceived inefficacy	17/149	11.41	20/115	17.39	37/264	14.02
Dissatisfaction	7/149	4.70	10/115	8.70	17/264	6.44
Nervous block	13/149	8.72	2/115	1.74	15/264	5.68
Insecurity	10/149	6.71	4/115	3.48	14/264	5.30
Disorientation	10/149	6.71	1/115	0.87	11/264	4.17
Lack of prior experience	2/149	1.34	8/115	6.96	10/264	3.79
Loss of control	8/149	5.37	2/115	1.74	10/264	3.79
Discomfort	3/149	2.01	6/115	5.22	9/264	3.41
Shame	0/149	0.00	7/115	6.09	7/264	2.65

*Emotions related to extrinsic factors*	*75/427*	*17.56*	*90/399*	*22.56*	*165/826*	*19.98*
Positives	52/75	69.33	70/90	77.78	122/165	73.94
Positive revaluation	5/52	9.62	36/70	51.43	41/122	33.61
Realistic immersion	18/52	34.62	11/70	15.71	29/122	23.77
Appreciation of the simulation	15/52	28.85	10/70	14.29	25/122	20.49
Feeling supported	8/52	15.38	7/70	10.00	15/122	12.30
Exceeding expectations	6/52	11.54	5/70	7.14	11/122	9.02
Negatives	23/75	30.67	20/90	22.22	43/165	26.06
Feeling observed	7/23	30.43	8/20	40.00	15/43	34.88
Lack of time	9/23	39.13	3/20	15.00	12/43	27.91
Feeling of difficulty	5/23	21.74	5/20	25.00	10/43	23.26
Influence of the simulated environment	2/23	8.70	4/20	20.00	6/43	13.95

*Note:* The table shows the number of times that each code was cited resulting from the analysis of each category concerning the total number of codes in each category or subcategory as appropriate. Stud.A1 = self-assessment during the simulation session. Stud.A2 = self-assessment during the video debriefing.

Abbreviations: c = code and TC = total codes.

## Data Availability

The data that support the findings of this study are available from the corresponding author upon reasonable request.

## Supporting Information

Supporting Information 1 Supporting file 1: TREND statement checklist.

Supporting Information 2 Supporting file 2: Post hoc pairwise comparisons communication and clinical observation strategies between students—peers and students—instructors.

Supporting Information 3 Supporting file 3: Post hoc pairwise comparisons of nonlistening attitude between students—peers and students—instructors.
