# Supplementary material for: The Impact of Video-Assisted Debriefing on Fostering Self-Critical Thinking in Mental Health Nursing Students
Source: J Nurs Manag. 2025 May 9;2025:5598639. doi: 10.1155/jonm/5598639 (PMC12084791; doi:10.1155/jonm/5598639)
Supplement: Supporting Information 2 — Supporting file 2: Post hoc pairwise comparisons communication and clinical observation strategies between students—peers and students—instructors. [file 5598639.f2.docx]

Supplementary material 2. Post-Hoc Pairwise Comparisons Communication and Clinical Observation Strategies.

| Pairwise Comparisons |  | Identifies themselves | Empathetic attitude | Manages the environmental context | Appropriate use of non-verbal language | Uses open questions | Practices active listening | Promotes responsibility | Explores psychopathological state | Explores lifestyle habits |
| --- | --- | --- | --- | --- | --- | --- | --- | --- | --- | --- |
| Stud.A1- Stud.A2 | W | 1.90 | 1.68 | -0.27 | 2.92 | 0.98 | 1.42 | 1.58 | -0.08 | 0.637 |
|  | p | .533 | .636 | .997 | .164 | .898 | .748 | .679 | 1.000 | .970 |
| Stud.A1-Peers | W | -9.67 | 7.41 | 6.92 | 7.40 | 7.33 | 4.05 | 7.13 | 54.90 | 3.62 |
|  | p | < .001* | < .001* | < .001* | < .001* | < .001* | .022* | < .001* | < .001* | .051 |
| Stud.A1-Instr | W | -10.47 | -7.75 | -4.53 | -2.70 | -5.98 | -8.38 | -9.84 | -82.89 | -9.66 |
|  | p | < .001* | < .001* | .007* | .223 | < .001* | < .001* | < .001* | < .001* | < .001* |
| Stud.A2-Peers | W | -11.84 | 3.73 | 7.94 | 4.94 | 6.81 | 2.30 | 3.98 | 65.42 | 2.55 |
|  | p | < .001* | .042* | < .001* | .003* | < .001* | .363 | .025* | < .001* | .271 |
| Stud.A2-Instr | W | -11.52 | -8.44 | -4.32 | -5.58 | -6.76 | -9.19 | -10.21 | -84.12 | -10.32 |
|  | p | < .001* | < .001* | .012* | < .001* | < .001* | < .001* | < .001* | < .001* | < .001* |
| Peers-Instr | W | -5.41 | -13.82 | -11.29 | -10.26 | -12.06 | -11.26 | -14.97 | -120.95 | -15.65 |
|  | p | < .001* | < .001* | < .001* | < .001* | < .001* | < .001* | < .001* | < .001* | < .001* |

*Note: Stud.A1= Self-assessment during the simulation session, Stud.A2 = Self-assessment during the video-debriefing, Instr =Instructor*
