# Supplementary material for: The Impact of Video-Assisted Debriefing on Fostering Self-Critical Thinking in Mental Health Nursing Students
Source: J Nurs Manag. 2025 May 9;2025:5598639. doi: 10.1155/jonm/5598639 (PMC12084791; doi:10.1155/jonm/5598639)
Supplement: Supporting Information 3 — Supporting file 3: Post hoc pairwise comparisons of nonlistening attitude between students—peers and students—instructors. [file 5598639.f3.docx]

Supplementary material 3. Post-Hoc Pairwise Comparisons of Non-Listening Attitude.

| Pairwise Comparisons |  | Ordering, directing or commanding | Threatening | Giving premature advice, suggestions or solutions | Persuading with logic, discussion, or teaching | Moralizing, preaching | Disagreeing judging and/or criticizing | Begging | Blaming, ridiculing, or labelling | Interpreting or analysing | Reaffirming, sympathizing, or consoling | Questioning or testing | Withdrawing, distracting, joking, changing the subject |
| --- | --- | --- | --- | --- | --- | --- | --- | --- | --- | --- | --- | --- | --- |
| Stud.A1- Stud.A2 | W | 0.388 | -0.733 | 0.252 | -0.102 | 0.336 | 0.0350 | 0.0301 | -0.121 | 0.712 | -0.0650 | 1.792 | 12.65 |
|  | *p* | .993 | .955 | .998 | 1.000 | .995 | 1.000 | 1.000 | 1.000 | .958 | 1.000 | .584 | < .001* |
| Stud.A1-Peers | W | 8.486 | 9.420 | 2.348 | 1.252 | 11.180 | 113.282 | 36.726 | 9.520 | 4.136 | 12.997 | 13.904 | 4.13 |
|  | *p* | < .001* | < .001* | .345 | .813 | < .001* | < .001* | .046* | < .001* | .018* | .795 | < .001* | .018* |
| Stud.A1-Instr | W | -1.754 | 1.064 | -9.445 | -5.255 | 5.762 | 29.879 | -59.190 | 2.488 | -7.882 | -106.710 | -0.329 | 2.08 |
|  | *p* | .601 | .876 | < .001* | .001* | < .001* | .149 | < .001* | .293 | < .001* | < .001* | .996 | .455 |
| Stud.A2-Peers | W | 7.435 | 9.791 | 1.068 | 2.156 | 10.441 | 107.463 | 37.594 | 9.170 | 3.702 | 17.201 | 10.687 | -7.81 |
|  | *p* | < .001* | < .001* | .875 | .423 | < .001* | < .001* | .039* | < .001* | .044* | .617 | < .001* | < .001* |
| Stud.A2-Instr | W | -2.132 | 1.739 | -9.542 | -5.086 | 5.198 | 28.628 | -60.904 | 2.536 | -8.388 | -103.743 | -2.036 | -9.82 |
|  | *p* | .433 | .876 | < .001* | .002* | .001* | .179 | < .001* | .277 | < .001* | < .001* | .475 | < .001* |
| Peers-Instr | W | -11.246 | -8.358 | -12.203 | -7.533 | -3.189 | -77.759 | -114.240 | -7.113 | -12.523 | -150.824 | -14.630 | -2.11 |
|  | *p* | < .001* | < .001* | < .001* | < .001* | .109 | < .001* | < .001* | < .001* | < .001* | < .001* | < .001* | .444 |

*Note: Stud.A1 =* *Self-assessment during the simulation session,* *Stud.A2 = Self-assessment during the video-debriefing, Instr =Instructor*
